# Intraoperative Tracheal Obstruction Management among Patients with Anterior Mediastinal Masses

**DOI:** 10.1155/2018/4895263

**Published:** 2018-07-04

**Authors:** H. Kafrouni, Joelle Saroufim, Myriam Abdel Massih

**Affiliations:** Anesthesiology and Pain Management, Saint George Hospital-University Medical Center, Beirut, Lebanon

## Abstract

**Background:**

Patients suffering from undiagnosed obstruction of the central airways: the trachea and main stem bronchi are at increased risk for perioperative and postoperative complications, especially if general anesthesia is performed.

**Case Description:**

This report discusses a 30-year-old asymptomatic Caucasian female who faced recurrent distal airway collapse during mediastinoscopy for biopsy of an anterior mediastinal mass, which led to the inability to extubate her. This case examines the necessity of a thorough preoperative assessment especially in patients with undiagnosed tracheal obstruction and a precise coordination between anesthesiologist and surgeon in being able to perform a safe and smooth anesthesia, in order to avoid life-threatening complications and to reduce further morbidity.

**Methods:**

The scope of this case report is restricted to publications in all surgical and anesthesiological specialties among adult patient population. Main search key words were as follows: “tracheal obstruction,” “general anesthesia,” “mediastinum,” and “tumors” *Results*. The literature supports an increased perioperative risk of airway obstruction with the use of general anesthesia in patients with anterior mediastinal masses. This case report suggests a perioperative anesthetic management modality for patients presenting with anterior mediastinal masses and who are at high risk of cardiovascular compression and tracheal obstruction. Thus, it is highly important to note that evidence-based recommendations are not available in the literature.

**Conclusions:**

This case report suggests perioperative management modalities performed by anesthesiologists in order to minimize the risk of airway obstruction among patients having anterior mediastinal masses and shed the lights on the importance of proper anesthetic and surgical planning in order to prevent intraoperative complications and improve the quality of healthcare provided to patients presenting critical cases.

## 1. Introduction

Mediastinal masses consist of a collection of benign and malignant tumors found in the mediastinum. Anterior mediastinal tumors (located in the anterior mediastinum) cause severe and life-threatening cardiorespiratory complications and even death. There are 7–18% incidence of airway obstruction of patients who were reported to have mediastinal masses and were under general anesthesia [1]. The risks of general anesthesia in patients with large anterior mediastinal masses have become well known. These tumors are difficult to manage in the preoperative period; it requires a multidisciplinary team to properly manage these patients having a central airway obstruction. The most common diagnosis of masses among adults are lymphoma (whether Hodgkin's or non-Hodgkin's), germ cell tumor, thymoma, bronchogenic carcinoma, granuloma, bronchogenic cyst, thyroid tumors, and cystic hygroma [2]. Clinical presentations of patients with mediastinal masses (anterior, posterior, middle, or superior) can be tracheobronchial obstruction, right heart and pulmonary vascular compression, superior vena cava syndrome, and other systemic syndromes such as myasthenia gravis or thyroid disease [3]. However, some patients can be asymptomatic, which is a challenge that healthcare professionals face especially if patients were not diagnosed and were intubated for general anesthesiology in a surgery. Children show earlier symptoms of airway obstruction than adults, due to smaller tracheal diameter and decreased cross-sectional area which leads to increased airway resistance [4].

Mediastinoscopy is a procedure indicated for diagnostic biopsy of a mediastinal mass or adenopathy, and it is required to assign appropriate treatment in accordance with the anatomopathologist result. General anesthesia is usually recommended, but in case of severe central airway compression, many anesthesiologists prefer local anesthesia in order to avoid unmanageable airway collapse [5].

## 2. Case Description

A 30-year-old Caucasian female consulted her physician after being subject to lethargy, night sweats, weight loss, and dry cough with dyspnea upon heavy exercise for one month. She was previously healthy, occasional smoker with no previous surgeries or any known allergies.

Series of lab tests showed a mild normocytic anemia with mild lymphocytosis, while a preoperative chest computed tomography scan, showed a 15 cm anterior mediastinal mass with no involvement of the adjacent structures: superior vena cava, pericardium, or pleura. Following that, the thoracic surgeon scheduled a diagnostic mediastinoscopy.

The implicated surgeon described the procedure to the anesthesiologist in charge of the patient as being risk-free, and that he needs general anesthesia due to the difficulty in accessing the mediastinal mass. Anesthetic induction was uneventful. The patient was easily ventilated and after reaching a proper anesthetic depth, she was intubated via uncomplicated direct laryngoscopy with a French endotracheal tube (ETT) 7.5 cm. Bilateral breath sounds were checked and even chest expansion were noticed. Pulse oximetry showed an arterial oxygen saturation of 99% with an end-tidal CO_2_ within the normal range.

Few minutes later, the patient developed a sudden airway collapse where end-tidal CO_2_ dropped significantly, and desaturation was noted with no chest expansion. The anesthesiologist directly extracted the tube, but the mask ventilation was unsuccessful; so he decided to immediately reintubate the patient to secure the airway. Then, the ventilation was regained, saturation increased, and CO_2_ curve reappeared. So as a result, airway collapse was reported and/or ETT displacement.

Later, the patient developed another airway collapse, so bronchospasm was suspected. Based on this suspicion, albuterol and Solu-Cortef were given. As a result, ventilation was regained, but with high inspiratory and positive end expiratory pressures and with an increase of the end-tidal CO_2_ to a critical level.

The anesthesiologist then performed a fiberoptic tracheal exploration, it showed a narrowed trachea just distal to the ETT due to an extrinsic mass obstructing the tracheal lumen and extending down the carina. Due to this new finding, he decided to postpone the procedure until the stabilization and the management of the airway stenosis. However, the surgeon insisted to proceed with this elective surgery.

Under diagnostic biopsy performance and mediastinoscope insertion, she developed another airway collapse while the patient was sedated, but ventilation was maintained at high inspiratory pressures and the patient was monitored for a consecutive of 15 minutes.

Biopsy from the anterior mediastinal mass was taken and sent to the anatomopathologist. Then, mediastinoscope was retracted, auscultation revealed bilateral minimal air entry. Propofol and sevoflurane used for maintenance of anesthesia were discontinued and 15 min later, the patient was breathing spontaneously but with some retractions. Upon giving Prostigmin, she developed another airway collapse with severe oxygen desaturation. The anesthesiologist decided to keep the patient intubated; she was transferred to the intensive care unit for observation and evaluation of her airway obstruction.

In the intensive care unit, thorough laboratory and radiology revealed a severe distal extrinsic tracheal compression and left main bronchus compression of more than 80% by the anterior mediastinal mass, with no involvement of the adjacent vascular structure. They also showed a left lower lobe consolidation in favor of a pneumonia and minimal bilateral pleural effusion. Meanwhile, she kept developing airway collapse with severe arterial oxygen desaturation.

She was treated with an empiric antibiotherapy, with intravenous steroids and nebulizers. A multidisciplinary team decided to begin chemotherapy after the anatomopathologist result came as a non-Hodgkin's lymphoma.

Subsequent to treatment, lymphoma size decreased progressively, relieving the airway compression, and extubation was performed after one week from undergoing the mediastinoscopy. After three days of observation, she was discharged home, with scheduled outpatient chemotherapy sessions and later possible surgical management.

## 3. Discussion

The case presented shows the importance of an appropriate communication between the anesthesiologist and the surgeon to generate a detailed preoperative and anesthetic assessment that could possibly prevent the complications that occurred.

The authors explained the primary mechanisms of inducing airway collapse in patients with tracheal obstruction when general anesthesia is applied. General anesthesia has been shown to affect pulmonary mechanics by reducing lung volume and functional residual capacity and by relaxing smooth airway muscles, thus increasing large airway compressibility. In addition, it has been found to have a partial obstructive respiratory effect and large negative pressures by flattening the trachea through extrinsic compression, muscular relaxation, and active airway inspiratory force disturbance, causing the chest wall volume and external support of narrowed airway to reduce. Other factors such as the supine position, the elimination of glottic regulation of airflow by endotracheal intubation, and the size and location of the mediastinal mass, as well as the existence of obstructive or restrictive preexisting airway diseases could contribute to the airway collapse [6].

Due to all these factors, many anesthesiologists prefer to avoid general anesthesia mainly in patients who have marked symptoms of airway compromise such as dyspnea at rest, postural dyspnea, orthopnea, or even stridor. Asymptomatic patients who present with tracheal stenosis of more than 50% of the residual cross area, or present with a pericardial effusion, or patients who are in favor of superior vena cava syndrome are at high risk of intraoperative airway problems. Mediastinoscopy can be done under local anesthesia, but in case a general one is required, a full and detailed patient assessment is essential in order to construct the safest anesthetic plan and to reduce risks [7].

### 3.1. Preoperative Assessment

All patients admitted for mediastinoscopy of an anterior mediastinal mass, should first of all do a preoperative chest X-ray and CT, as they will provide information concerning the site, severity, and the extent of any existing mediastinal mass [5]. Flow-volume loops and pulmonary function test help in the diagnosis of associated abnormalities in lung function [5].

Patients at high risk of airway obstruction intraoperatively may benefit from pretreatment of the mediastinal mass with steroids, empiric chemotherapy, and/or radiotherapy [5]. This treatment can cause rapid tumor lysis and alleviate airway obstruction but also may adversely affect the accuracy of tissue diagnosis once a biopsy is taken. A study done by Bechard et al. report that it is preferable to acquire a tissue diagnosis before starting treatment, even if doing so necessitates the use of general anesthesia, except in extreme circumstances [8].

### 3.2. Premedication

Sedation should be avoided, if a potential airway collapse could occur. Otherwise, midazolam 1-2 mg intravenous may be appropriate. Antisialogogue, can be given if an awake intubation is decided [7].

### 3.3. Anesthetic Management

Patients with tracheal stenosis can suffer from sudden cardiorespiratory arrest when receiving general anesthesia, especially with patients with 50% obstruction of airway at the level of the lower trachea and main bronchi [7]. Some studies have recommended that these patients have their femoral veins cannulated in readiness for a cardiopulmonary bypass, as a preventive step in case of a cardiorespiratory arrest [3].

In the operating room, a large bore intravenous cannula is inserted and an arterial line is instituted for an invasive arterial blood pressure monitoring, for the early detection of reflex arrhythmias and compression of major vessels during mediastinoscopy.

A 20-degree head up position is preferred to reduce mass compression effect on airway, vascular structures, and venous congestion.

Induction of anesthesia can be done with intravenous and/or inhalational anesthetic agent. If a muscle relaxant is required, assisted ventilation should first be done manually, to assure that positive-pressure ventilation is possible and only then can a short-acting muscle relaxant be administered [5, 7]. A bolus or continuous infusion of a short-acting opioid will allow an adequate level of anesthesia and a rapid postoperative recovery. Ventilation of both lungs through a single-lumen endotracheal tube is usually adequate. A reinforced tube is preferred to minimize the risk of the tube kinking during surgery [7].

A surgeon familiar with rigid bronchoscopy should be available in the operating room during induction of general anesthesia, allowing immediate tracheal obstruction management.

In summary, patient suffering from central airway compression by an anterior mediastinal mass requires a preoperative anesthetic planning in coordination between the anesthetist and the surgeon in charge, in order to prevent possible perioperative and postoperative complications [6].

## References

[1] Thaur P., Bhatia P. S., Vimani P. (2014). Anaesthesia for mediastinal mass. *Indian Journal of Anesthesia*.

[2] Slingera P., Karslib C. (2007). Management of the patient with a large anterior mediastinal mass: recurring myths. *Current Opinion in Anaesthesiology*.

[3] Ku C. M. (2011). Anesthesia for patients with mediastinal masses. *Principles and Practice of Anesthesia for Thoracic Surgery*.

[4] Datt V., Tempe D. K. (2005). Airway management in patients with mediastinal masses. *Indian Journal of Anaesthesia*.

[5] Hammer G. B. (2004). Anaesthetic management for the child with a mediastinal mass. *Paediatric Anaesthesia*.

[6] Li W. W., Boven W. J., Annema J. T., Eberl S., Klomp H., De Mol B. (2016). Management of large mediastinal masses: surgical and anesthesiological considerations. *Journal of Thoracic Disease*.

[7] Ng A., Bennett J., Bromley P., Davies P., Morland B. (2007). Anaesthetic outcome and predictive risk factors in children with mediastinal tumours. *Pediatric Blood and Cancer*.

[8] Bechard P., Letouneau L., Lacasse Y., Cote D., Bussieres J. (2004). Perioperative cardiorespiratory complications in adults with mediastinal mass: incidence and risk factors. *Journal of the American Society of Anesthesiologists*.

